# A Clinical Case Report of Deficiency of Adenosine Deaminase 2 Syndrome (DADA 2) Presenting as a Brachial Artery Aneurysm

**DOI:** 10.31138/mjr.120324.dai

**Published:** 2024-10-23

**Authors:** Shama Sowdagar, Avanish Jha, Prabhu Vasanth, John Mathew

**Affiliations:** Department of Clinical Immunology and Rheumatology, Christian Medical College, Vellore, Tamil Nadu, India

**Keywords:** DADA 2 syndrome, vasculitis, peripheral arterial aneurysm, brachial artery, polyarteritis nodosa

## Abstract

**Introduction:**

Deficiency of adenosine deaminase 2 (DADA 2) syndrome is a monogenic auto-inflammatory vasculitic syndrome caused by loss of function mutations in the ADA2 gene. Disease manifestations are divided into three major phenotypes: inflammatory/vascular, immune dysregulation, and haematologic, with majority having significant overlap between these phenotypes. The disease has undergone extensive phenotypic expansion since its first description in 2014. It is autosomal recessively inherited and commonly presents with fever, recurrent strokes, livedo racemosa, and polyarteritis nodosa (PAN)-like features. Though the disease has its symptom onset early in childhood, various case series have described patients with symptom onset in adulthood. Visceral arterial aneurysms as a manifestation have been described in literature but not peripheral aneurysms. Here, we describe a young adult with DADA 2 syndrome presenting with peripheral arterial aneurysm involving the brachial artery.

**Case report:**

Patient had recurrent episodes of CVA since childhood, a history of orchitis, systemic hypertension, and new onset brachial artery aneurysm at the age of twenty-three. Diagnosis was confirmed by genetic analysis. Tumour necrosis factor inhibitors (TNFi) have emerged as the drug of choice for the treatment of DADA2 and studies revealed a drastic reduction in stroke risk after initiation of TNFi. Based on this experience, we have started the patient on adalimumab, and he is doing well for the past one year.

**Conclusion:**

Peripheral artery aneurysm can be a manifestation of vasculitis in DADA 2 syndrome.

## INTRODUCTION

DADA 2 syndrome is a recently recognised monogenic auto-inflammatory condition with phenotypic heterogeneity in terms of age of onset, symptoms, organ involvement, and severity.^1^ It has been reported to respond better to tumour necrosis factor inhibitors than traditional immunosuppressive agents.^2^ Aneurysms involving the visceral arteries have been reported in various case series of DADA 2.^3,4^ We report the first case of DADA 2 syndrome presenting with a peripheral arterial aneurysm involving the brachial artery.

## CASE PRESENTATION

**Figure 1.**
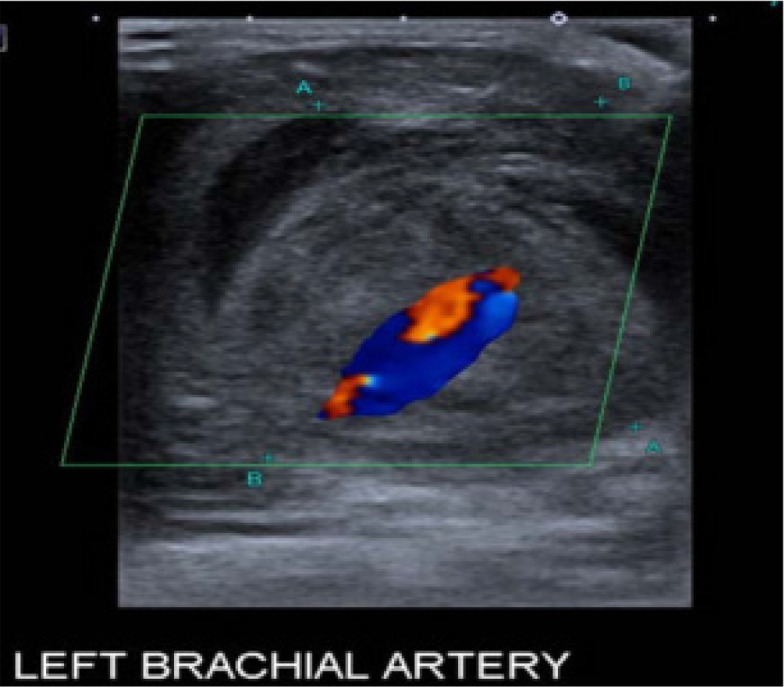
Colour Doppler ultrasound showing the classic yin-yang flow pattern within the aneurysm originating from brachial artery.

Mr. A, 24-year-old young male, born to 3^rd^ degree consanguineous married couple, was referred to the rheumatology OPD with a history of painless progressive swelling in the left arm for 2 months duration. There was no history of trauma, intravenous drug use, or procedure prior to the onset of swelling. He had an episode of cerebrovascular accident (CVA) at the age of 8 years and was started on single antiplatelet agent. He was later switched to dual antiplatelet therapy following a recurrence of CVA. He remained quiescent until 21 years of age, when he developed recurrent episodic right scrotal pain with testicular tenderness for which he underwent orchidopexy. He developed new-onset systemic hypertension at the age of 23 years for which he underwent evaluation and was initiated on anti-hypertensive medication. He developed a swelling in the left arm six months later, which was suggestive of a brachial artery aneurysm on Colour Doppler ultrasound (**Figure 1**). Blood reports were normal except for mild anaemia and an elevated C-reactive protein (CRP). In view of recurrent episodes of CVA since childhood, history of orchitis, systemic hypertension, family history of consanguinity, and a new-onset brachial artery aneurysm with elevated inflammatory markers, we considered the possibility of DADA 2 syndrome.

## INVESTIGATIONS

His past records revealed elevated erythrocyte sedimentation rate (ESR), lacunar chronic infarcts on imaging, normal ECHO, negative pro-thrombotic work up, and a negative sickling test done at 8 years of age. A brief work up done for common secondary causes of vasculitis were negative for Anti-nuclear antibodies (ANA), Rheumatoid factor, Anti neutrophil cytoplasmic antibodies (ANCA) and antiphospholipid antibodies. Extensive work up for cardiac, endocrine, renal and vasculitis aetiologies were normal including CT angiography done at time of diagnosis of hypertension. However, he had never had a conventional angiogram done till date. The excision biopsy of the aneurysm showed blood vessel containing organising thrombus with loss of intima, moderate to dense inflammatory infiltrates in the media, and marked fibrosis of the media. Adventitia also had proliferated and congested blood vessels with overall features suggestive of vasculitis. There was no evidence of granuloma or multinucleated giant cells. In view of strong suspicion of DADA 2 syndrome, he underwent a genetic testing with clinical exome sequencing for ADA2 gene. It showed homozygous pathogenic missense mutation (Gly47Arg) in exon 2 confirming the diagnosis DADA 2 syndrome (**Table 1**).

**Table 1.** Clinical exome sequencing report of the patient showing homozygous mutation involving ADA 2 gene.

**Gene (transcript)**	**Location**	**Variant**	**Zygosity**	**Disease (OMIM)**	**Inheritance**	**Classification**
ADA 2 (-)	Exon 2	c.139G>A (p.Gly47Arg)	Homozygous	Sneddon syndrome/Vasculitis, auto inflammation, immunodeficiency, and haematologic defects syndrome	Autosomal recessive	Pathogenic

## RESULT

Pathogenic variant causative of the reported phenotype was detected.

## TREATMENT

We started him on oral steroid while awaiting genetics report. He was later initiated on TNF inhibitor therapy with adalimumab and steroid was gradually tapered and discontinued. He has been thriving without issues for the past year. His antiplatelet medications were discontinued due to the risk of bleeding associated with DADA2.

## DISCUSSION

Our case highlights a new presentation of DADA 2 syndrome as peripheral arterial aneurysm involving the brachial artery and adds on to the expanding manifestations of this recent monogenic auto inflammatory syndrome and to the spectrum of DADA 2 syndrome reported from our centre.^7^

DADA 2 syndrome is an autosomal recessive monogenic auto inflammatory disease, a mimicker of a primary systemic vasculitis - polyarteritis nodosa (PAN). It is caused by a biallelic loss of function mutation in ADA 2 gene.^1^ The clinical phenotype is significantly broadening which includes vascular pathology with ischemic and haemorrhagic strokes, livedo racemosa, systemic inflammation w ith r ecurrent fevers, portal and systemic hypertension, haematological abnormalities like pure red cell aplasia and immunodeficiency. TNF inhibitors have emerged as the drug of choice for the treatment of DADA2 for the vasculitic phenotype though there are no randomised controlled trials available.^4^ In severe disease with haematological involvement or immunodeficiency, l ong-term s uccess with allogenic hematopoietic stem cell transplantation has been reported.^3, 6^

Literature review of DADA 2 syndrome showed evidence of various visceral artery microaneurysms; however, there is no description of peripheral aneurysm involving the brachial artery (**Table 2**). Brachial artery aneurysm itself is a rare upper extremity aneurysm that typically occurs because of trauma or infection or puncture by procedures.^5^ Our patient did not have any of these risk factors. Our patient had childhood onset of symptoms under 10 years of age with recurrent ischemic strokes however, there was a delay in diagnosis until 24 years. He developed orchitis, systemic hypertension, and brachial artery aneurysm in the 3rd decade of his life. Missense mutations with residual enzymatic activity align with the vasculitis phenotype and later presentations as in our case. Our patient had pathogenic homozygous missense mutation presenting as vasculitic phenotype with systemic inflammation and thus treated with TNF inhibitor. Variants with minimal residual function, including nonsense mutations and insertion/deletion mutations, are associated with severe haematologic compromise and immuno-deficiency and these features were not present in our patient. Anti-TNF therapy controls the inflammatory manifestations preventing the occurrence of vascular events in patients.^2^ Antiplatelet agents and anticoagulants are controversial, as they may increase the risk of haemorrhagic strokes.^3^ Hence, we discontinued antiplatelet agents once the diagnosis was confirmed.

**Table 2.** Analysis of cases of DADA2 with aneurysms.

**S. no**	**Age/Sex (Years)**	**Type of aneurysm**	**Age of symptom onset (Years)**	**Age at diagnosis (Years)**	**Clinical presentation**	**ADA2 gene mutation**	**Treatment**	**Duration of follow-up and outcome**
1. Current case report	24/M	Brachial A. aneurysm	8	24	Recurrent CVA Testicular pain HTN	G47R	Steroid Adalimumab Excision biopsy of aneurysm	Good clinical response after 1 year follow up on treatment
2. Geraldo AF et al.^8^ 2021	18/F	Intracranial A. aneurysm	5	17	Livedo reticularis Skin ulcers Arthralgia Recurrent strokes	G47R	Steroid Etanercept	Complete clinical response at 19 years age
3. Navon Elkan P et al.^9^ 2014	23/M	Coronary artery aneurysm	2 months	18 months	Fever Livedo reticularis Cutaneous necrosis HTN GI involvement	G47R	Steroid Cyclophosphamide Infliximab Azathioprine Aspirin	Good response Continues therapy at 11 years follow up
	33/F	Mesenteric and hepatic A. aneurysm	5	17	Myalgia Livedo reticularis Skin nodules GI involvement Panniculitis	G47R	Steroid Etanercept	Remission continues therapy at 18 years follow up
	29/M	Intracranial A. aneurysm	7 months	16	Fever Livedo reticularis Skin nodules Recurrent stroke HTN	G47R	Steroid Cyclophosphamide Etanercept Methotrexate	17 years follow up Continues therapy Good response Occasional mild cutaneous exacerbations
	38/M	Renal and mesenteric A. aneurysm	10	10	Fever Myalgia Livedo reticularis HTN GI involvement Necrotising vasculitis	Compound heterozygous for G47V T264S	Steroid Azathioprine Cyclophosphamide Aspirin	17 years Good response Continues systemic therapy
	28/F	Renal and SMA aneurysm	1	-	Fever HTN GI involvement CNS encephalopathy	G47R	Steroid Cyclophosphamide Infliximab	Recently diagnosed Good response to infliximab
	2m/F	Coeliac and renal A. aneurysm	2 months	7 months	Fever GI involvement Digital necrosis CNS- Ventricular Haemorrhage	G47R	Steroid Cyclophosphamide	Died at 9 months age
	36/M	Renal A. aneurysm	16	-	Raynauds Multiple leg ulcers Livedo reticularis Testicular pain HTN	G47R	Steroid Cyclophosphamide	20 years follow up, poor compliance to treatment Severe hypertension
4. Nanthapisal S et al.^10^ 2016	24/F	Renal A. aneurysm	6	24	Livedo racemosa Digital ischemia Lymphopenia HTN Intracerebral haemorrhage	Compound heterozygous 506C>T R169G	Steroid Cyclophosphamide Azathioprine Mycophenolate mofetil Infliximab	Not described
5. Belot A et al.^11^ 2014	6/M	Micro aneurysms involving hepatic, renal, and SMA	3 weeks	6	Arthritis Recurrent fevers Skin nodules Recurrent CVA Cranial neuropathies	G47R	Steroid Mycophenolate mofetil Cyclophosphamide	No detailed description Steroid dependent
6. Agajany N et al.^12^ 2023	42/F	Middle and anterior cerebral A. aneurysms	Since childhood	42	Recurrent limb ulcers Livedo reticularis Sensorineural hearing loss Recurrent episodes of amaurosis fugax	G47R	Anti-TNFi	Good response at 10 months follow up

## CONCLUSION

- Peripheral artery aneurysm can be a manifestation of vasculitis in DADA 2 syndrome.
- A high index of suspicion of DADA 2 syndrome is required in those who present with PAN-like features in either childhood or adults with predominant CNS manifestations.

## CONFLICT OF INTEREST

The authors declare no conflict of interest.
